# Seed size variation impacts local adaptation and life-history strategies in a perennial grass

**DOI:** 10.1098/rspb.2022.2460

**Published:** 2023-05-10

**Authors:** Samsad Razzaque, Robert W. Heckman, Thomas E. Juenger

**Affiliations:** Department of Integrative Biology, The University of Texas at Austin, Austin, TX 78712, USA

**Keywords:** ecotypic differentiation, life-history evolution, local adaptation, trade-off

## Abstract

Seed mass is an ecologically important trait that often differs considerably among ecotypes. Yet, because few studies examine the impacts of seed mass on adult life-history traits, its role in local adaptation is unclear. In this study, using accessions of *Panicum hallii* that spanned the two major ecotypes, we examined whether covariation between seed mass, seedling and reproductive traits impacts ecotypic divergence and local adaptation. The perennial grass *P. hallii* has two distinct ecotypes—a large-seeded upland ecotype adapted to xeric environments and a small-seeded lowland ecotype adapted to mesic environments. In the greenhouse, seed mass varied greatly across *P. hallii* genotypes in a manner consistent with ecotypic divergence. Seed mass covaried significantly with several seedling and reproductive traits. At field sites representing the habitats of the two ecotypes, seed mass had different impacts on seedling and adult recruitment: selection favoured large seeds in upland habitat and small seeds in lowland habitat, which was consistent with local adaptation. By demonstrating the central role of seed mass in ecotypic differences in *P. hallii* and its importance to seedling and adult recruitment under field conditions, these studies show that early life-history traits can promote local adaptation and potentially explain ecotype formation.

## 1. Introduction

Because plants move as seeds, seed traits are important determinants of population dynamics, including survival in current habitats and colonization of new habitats [1,2]. One seed trait that is critical to population dynamics is seed mass. Seed mass varies enormously across species and is generally thought to be under stabilizing selection [3,4]. Moreover, seed mass is a key determinant of plant reproductive strategies that has important ecological consequences. For example, larger-seeded plants often germinate, survive and grow at higher rates than smaller-seeded plants [5,6]. Seed mass may also impact adult and reproductive traits [7,8], but few studies in natural populations have observed this so far [9,10]. Thus, there is an important gap in our understanding of how seed and seedling characteristics affect adult success [10,11] and subsequent population dynamics.

Seed mass may play a crucial role in adaptation within a species's geographical range [12,13]. One hypothesis states that habitat quality and local competitive environments are major selective forces driving the evolution of seed and seedling phenotypes [14,15]. Broad surveys have found associations between seed size and environmental conditions, suggesting that variation in seed size may be adaptive [16,17]. For instance, dry environments often favour larger seeds [16]. This may occur because larger-seeded species can provision more resources to increased root growth, which increases access to soil water [18,19]. Environmental differences may also drive the evolution of seed size within species. If seed size has different impacts on establishment in contrasting habitat types (e.g. mesic versus xeric), this could promote ecotype formation [20,21]. Although the role of seed size in ecotype formation is unresolved, one way to test this hypothesis is to conduct a reciprocal field experiment across sites with substantial environmental differences [22,23].

Ecotypic divergence often results from trade-offs where, given finite resources, adaptation to one environment results in a fitness cost in alternative environments [24,25]. For instance, adaptation to xeric versus mesic habitats often promotes differences in traits related to water status maintenance and drought tolerance [26,27]. Similarly, a trade-off may occur between provisioning resources to individual seeds and seed dispersal ability [28,29]. This trade-off could explain why large-seeded plants often produce fewer seeds [30,31] and exhibit more rapid early growth and establishment than small-seeded plants [32]. Moreover, trade-offs can occur at different levels of organization (e.g. individual, population and ecotype) within species [24]. Locally adapted populations often experience different selective pressures, which could lead to different trait relationships in their home environments. But, trait relationships that emerge among locally adapted populations may not be the same as those that emerge among genotypes within populations [33]. Given the potential importance of seed size for adaptation to abiotic stress gradients, it is critical to understand how the relationship between seed size and other life-history traits changes across levels of organization.

Here, we studied the strong ecotypic divergence in seed mass, early growth and adult life-history strategies in the C_4_ perennial bunchgrass *Panicum hallii* Vasey [34]. *Panicum hallii* is distributed across a wide climatic gradient in North America. The species consists of two well-characterized ecotypes—upland (var. *hallii*) and lowland (var. *filipes*)—that differ in seed size and other life-history traits. To date, ecotypic divergence in *P. hallii* has been studied primarily by comparing a representative upland genotype (HAL2) and a representative lowland genotype (FIL2) [34–36], which limits our ability to understand life-history trade-offs across levels of organization. But certain environments within the distribution of *P. hallii* might select for different trait combinations or strategies to suit their local conditions better. Thus, we cannot generalize the pattern of ecotypic divergence in *P. hallii* from two representative genotypes.

In this study, we explored seed-based life-history strategies in the greenhouse and field that incorporated the range of natural variation in *P. hallii*. This allowed us to answer the following five major questions. (1) How is phenotypic variation in life-history traits arrayed across different levels of genetic variation (e.g. individuals, populations and ecotypes)? (2) Is climate of origin a good predictor of seed size? (3) Are there ecotype-specific patterns of covariation between seed size and other life-history traits? (4) Are ecotypes locally adapted to sites that represent their xeric and mesic origins? (5) How strong is the selection on seed size in xeric and mesic habitats?

## 2. Material and methods

### (a) Study system, ecotypic divergence and population structure

*Panicum hallii* is native to North America, ranging from northern Mexico to southern Oklahoma and from Louisiana to Arizona. Across its range, temperature, precipitation and elevation vary considerably. Mean annual temperature ranges from 10.5°C to 23.9°C. Elevation varies from sea level to over 2200 m. Annual precipitation changes by more than 100 cm [37]. *Panicum hallii* has two major ecotypes: a lowland ecotype (*Panicum hallii* var. *filipes*) adapted to mesic coastal habitats, including coastal prairies along the Gulf coast of Mexico and Texas and the Rio Grande Valley, and an upland ecotype (*Panicum hallii* var. *hallii*), which is more widely distributed, and adapted to xeric portions of the southwestern US. These differences in distribution and habitat type—including greater than 1M years of evolutionary divergence [34]—have also led to large differences in ecology: upland plants are smaller in stature and occur in sparsely vegetated areas; lowland plants are larger and often grow in densely vegetated coastal prairies with other grasses (e.g. *Bouteloua rigidiseta*, *Nassella leucotricha*, *Hilaria belangeri*, *Bouteloua dactyloides*) [34,35].

Considerable genetic population structure exists in *P. hallii*. Palacio-Mejía *et al*. [37] identified seven genetic clusters that correspond with major ecoregions. Despite its narrow natural distribution, *var. filipes* was grouped into three genetic clusters and showed greater genetic diversity (H_e_) than the more widespread var. *hallii*. The var. *hallii* was also grouped into three genetic clusters. A seventh genetic cluster occurred in south Texas where both varieties coexist under sympatric conditions (Sympatric). Individuals from the sympatric cluster were admixed and showed evidence of gene flow between var. *hallii* and var. *filipes* [37]. Because this cluster showed intermediate phenotypic and genetic characteristics, we considered it a distinct ecotype (electronic supplementary material, figure S1 and file S1). Inclusion of the sympatric cluster as an admixed ecotype highlights that the ecotypes we study are not reproductively isolated and that gene flow has occurred between these divergent groups. Including this cluster also shows the impact of recombining functional trait sets on plant performance.

### (b) Plant materials, seed bulking and seed mass measurement

We characterize the natural diversity of *P. hallii* by studying plants from 123 distinct localities (electronic supplementary material, figure S1 and file S1). To do this, we bulked seeds for the entire diversity panel (one maternal genotype per collection locality) to avoid maternal effects and timing biases of seed collection. From field observation and previous data, we assume that *P. hallii* is largely selfing in the field—it has very low heterozygosity—and as such, each maternal line can be considered a homogeneous inbred line [34,38]. We bulked seeds in growth chambers located at the University of Texas at Austin. For seed bulking, seeds were germinated directly in a 3.5-inch pot in the spring of 2018. Soils were prepared by mixing potting medium (Pro Mix BX Mycorise PRO), coarse expanded shale (Turface MVP), and fine expanded shale (Turface Profile) at a ratio of 6 : 1 : 1. Plants were watered from the bottom every 3 days until seed collection. Growth chambers were set at 28°C with a 12 h light/12 h dark photoperiod. We inspected panicles regularly to ensure that seeds were collected when fully ripe. Seed quality for subsequent experiments was ensured by separating good seeds from chaff, detritus or unripe seeds. Good seeds were kept in coin envelopes at room temperature for six months, then weighed to calculate seed mass. We weighed 100 seeds per genotype using an analytical balance (Mettler Toledo, Columbus, OH, USA) in three independent sets. Average seed mass was calculated for each genotype by dividing total mass by 100 (seed mass = mass of 100 seeds ÷ 100). These seeds were used to estimate genotype-level seed mass; they were also used to grow plants for the greenhouse and field studies described below.

### (c) Greenhouse study to measure seedling, adult and reproductive traits

We initiated a greenhouse study at the University of Texas at Austin using seeds collected from the seed bulking in the fall of 2018. We germinated seeds in Petri dishes (25 mm × 100 mm) containing 60 g of sterilized sand and 13 ml of tap water. We sprayed approximately 1 ml of water on the lid of the Petri dish, then sealed it with parafilm to maintain moisture. The study was replicated across three temporal blocks; each block contained a single Petri dish (our unit of replication) of each genotype. Each Petri dish contained 15 seeds. Thus, the entire experiment comprised 369 Petri dishes: 123 genotypes × 3 replicates. Once germinated, we kept seedlings in Petri dishes for 18 days, then transferred three seedlings from each petri dish to a 3.5-inch pot. We thinned pots to a single seedling 20 days after transfer. Each pot contained the soil mix described above, was watered from the bottom every 3 days, and the position of each pot was randomized between trays every 10 days.

We measured several seed, seedling and adult traits in this study. First, we measured early growth traits on seedlings in Petri dishes. We inspected Petri dishes daily between 11.00 and 14.00 to record the first germinated seedling (germination time). At 18 days after the first seedling in each dish germinated, we counted the proportion of seedlings that had germinated (germination percentage) as a proxy for seed dormancy. We measured root and shoot length of three representative seedlings per genotype at 18 days after the first seedling in each dish germinated. Both root and shoot were fully outstretched before measurements were taken. Then, after transplanting seedlings into 3.5-inch pots, we measured adult traits. We inspected pots daily to record the date at which the first panicle was visible (flowering time). We also counted the total number of seeds produced by each panicle (seed number). In a previous study, we observed that ripened seeds shattered quickly, resulting in a loss of approximately 70% of seeds (S.R. & T.E.J. 2019, unpublished data). Consequently, we counted seed number before seeds were fully mature (15 days after flowering time). Finally, we measured aboveground biomass on senesced plants that had been dried for 72 h at 50°C (see electronic supplementary material, table S1 for a brief description of phenotypes measured and the data are provided at electronic supplementary material, file S1). We measured adult traits from a single bout of reproduction. Given that *P. hallii* often continues to produce new vegetative and reproductive biomass under benign greenhouse conditions rather than senescing, we attempted to match the experimental duration to the natural phenology of *P. hallii*, which we observed at three field sites (Brackenridge Field Laboratory and Lady Bird Johnson Wildflower Center in Austin, TX; Nueces Delta Preserve in Odem, TX). Specifically, we initiated the experiment in the greenhouse when *P. hallii* emerged at these field sites and withheld watering in the greenhouse when *P. hallii* had fully senesced at these field sites. Importantly, all experimental plants had flowered and produced seed before we ceased watering them. We used all life-history data collected in this experiment to estimate the pattern of ecotypic divergence (question 1) and covariation between seed mass and other life-history traits (question 3) in *P. hallii*.

### (d) Reciprocal field experiment to measure seedling and adult recruitment

We performed a field experiment to study the performance of genotypes at two sites that were representative of the xeric and mesic habitats occupied by *P. hallii*. The xeric site was at Brackenridge Field Lab (31.61703°N, −97.89774°W; Austin, TX, USA), and the mesic site was at Nueces Delta Preserve (27.92422°N, −97.61660°W; Odem, TX, USA). We established the experiments in areas where *P. hallii* occurred naturally. Our experiment included three genotypes from every population cluster defined by Palacio-Mejía *et al*. [37]. At each site, we added 100 seeds of a randomly assigned genotype to each 0.5 m × 0.5 m plot. Each genotype was replicated seven times at each site, for a total of 294 plots (21 genotypes × 7 replicates × 2 field sites). We also included seven control plots at each site to which no seed was added. Control plots were used to estimate seedling recruitment from the natural seed bank. Plots were not watered or disturbed. All plots were inspected every 15 days from seed addition in early February 2019 until December 2019. To do this, we identified visible seedlings in each plot and marked them with a rubber band to follow them through early development and flowering. Tagging each seedling allowed us to track plants until they flowered. From these observations, we calculated the number of germinated seedlings per plot and the number of seedlings that reached the adult stage (electronic supplementary material, file S2). From this field experiment, we assessed the pattern of seedling and adult recruitment for genotypes that differed in seed size (question 4). We also quantified natural selection on different seed size strategies in xeric and mesic habitats (question 5).

### (e) Statistical analysis

To obtain a multidimensional overview of life-history trait variation and integration (question 1), we performed linear discriminant analysis (LDA). We performed LDA on genotype-level means of seed, seedling and adult traits measured in the greenhouse study (multivariate function, JMP Pro 15, SAS, Cary, NC).

To calculate the proportion of variance in seed, seedling, and adult traits that was explained by ecotype, population groups and genotype (question 1), we used linear mixed effects models (lmer() function in lme4 [39]) in R [40]. Each model included the nested random effects of genotype within population cluster within ecotype. To estimate 95% confidence intervals, we performed 1000 bootstrap iterations (bootstraps() function in rsample [41]). Because the lowland ecotype had a very few genotypes, we condensed the three lowland population clusters into a single group.

We evaluated the effects of climate-of-origin on seed mass (question 2) by performing model selection using a linear mixed effects model (lme() function in nlme [42]). To do this, we built a global model that included seven continuous fixed effect predictors—elevation, latitude, longitude, mean annual temperature, temperature seasonality, annual precipitation and precipitation seasonality—and a random effect of genotype nested within population cluster within ecotype. We obtained historical climate data from each collection location from the Worldclim database [43] at 2.5 min spatial resolution. We ran all possible additive combinations of these seven predictors (dredge() function in MuMIn [44]), then performed model averaging (model.avg() function in MuMIn [45]) on all candidate models for which ΔAICc < 7. Because climate variables were often correlated with one another and with geography, this approach allowed us to evaluate the impact of individual climate variables on seed mass while limiting confounding between predictors.

Because seed mass is hypothesized to be an important driver of life-history strategies in *P. hallii*, we assessed the relationships between seed mass and other seed and seedling traits (question 3) using standardized major axis (SMA) regression (sma() function in smatr [46]). SMA regression assumes that both traits are measured with error. Thus, it is most appropriate for examining relationships between variables with an unclear causal relationship [47]. For each model, we regressed seed mass against one other trait (measured in the greenhouse study) and allowed this relationship to vary by ecotype (three levels).

We analysed field recruitment in two ways. First, we estimated the impact of ecotype and site on seedling recruitment (total seedlings in a plot) and adult persistence (total adults in a plot) by fitting a generalized linear model with a Poisson distribution and log link (question 4). These models included seedling or adult count as the response and ecotype, site and their interaction as categorical predictors. We determined that the Poisson GLM adequately fitted the data using the Pearson goodness of fit statistics (JMP v. 15.1.0, SAS Institute, Cary, NC).

Next, we estimated the strength of selection on seed mass under differing field conditions (question 5) by fitting a multivariate hierarchical Bayesian model (Stan [48]; brms [49]). This model included two correlated responses—the number of seedlings per plot and adults per plot. Seedling number was predicted by fixed effects of genotype-level seed mass, site identity and their interaction; adult number was predicted by seedling number, genotype-level seed mass, site identity and their interactions. Each response also had two random effects—plot ID and genotype nested within population clusters within ecotype; plot ID was correlated between response variables to account for the non-independence between adult and seedling counts. Both responses were modelled with a Poisson distribution and a log link. Models had weakly informative priors: for fixed effects, we used *N*(0, 25); for intercepts, we used *N*(0, 10); and for standard deviations on random effects, we used the brms default priors, student-*t*(3, 0, 10)^1^. Using more diffuse priors on fixed effects did not qualitatively change the results. This model included three chains with 4000 iterations each. Chains converged on a stationary distribution based on trace plots and model diagnostics (Rhat = 1). Models adequately fitted the data based on visual assessment (pp_check() function).

## 3. Results

### (a) Life-history traits show patterns of ecotypic divergence in multivariate space

**Figure 1.**
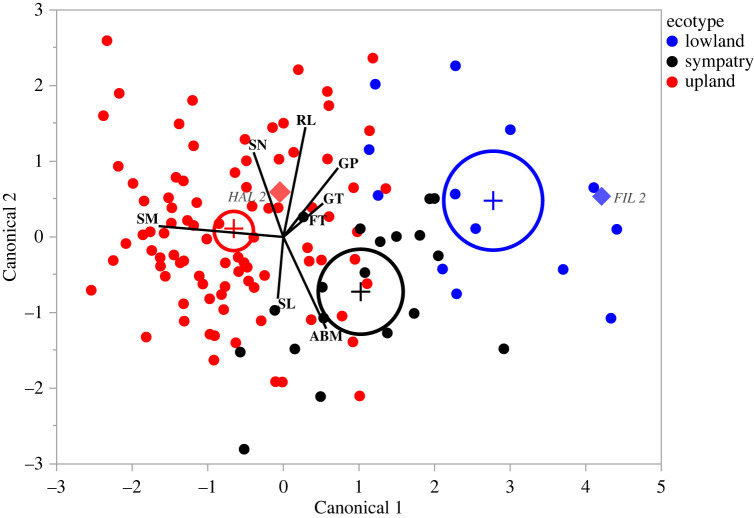
Linear discriminant analysis of seed, seedling and reproductive traits in *Panicum hallii.* Points represents the means of individual genotypes. The lowland ecotype is blue, the sympatric ecotype is black, and the upland ecotype is red. Each ecotype's multivariate mean is denoted by a ‘+’ marker. 95% confidence level ellipses are plotted for each ecotype mean. The genome reference lines of the upland genotype (HAL2) and lowland genotype (FIL2) are marked as ‘♦’ in the plot. Traits: SM, seed mass; SL, shoot length; RL, root length; GT, germination time; GP, germination percentage; FT, flowering time; ABM, aboveground biomass; SN, seed number.

We conducted linear discriminant analysis (LDA) on genotype-level trait means from the greenhouse study to explore the multivariate structure of seed, seedling, and adult traits in *P. hallii* (question 1). The lowland and upland ecotypes diverged substantially based on genotype-level trait means (figure 1). Specifically, the first canonical axis explained 92% of differentiation among the ecotypes (*p* < 0.0001). Seed mass had the highest loading on canonical axis 1, suggesting that variation in seed mass was strongly associated with ecotypic divergence. The representative genotypes, HAL2 and FIL2, differed considerably from one another in multivariate space, but were not entirely representative of their respective ecotypes: HAL2 occurred toward the middle of the space occupied by upland genotypes and FIL2 was at an extreme of the space occupied by lowland genotypes (figure 1). This suggests that there is additional variation—including at very large seed size—that is not accounted for when studies focus on HAL2 and FIL2.

Linear discriminant analysis (LDA) accurately classified 77% of individuals to ecotype. Classification accuracy differed among ecotypes in an informative way: the upland and lowland ecotypes were correctly classified 80% and 78% of the time, respectively, while the sympatric population was only accurately classified 63% of the time. Moreover, only 2% of the upland individuals were misclassified as lowlands, and none of the lowlands were misclassified as uplands. On the other hand, 10% of sympatric individuals were classified as uplands and 26% were classified as lowlands.

### (b) How is phenotypic variation in life-history traits arrayed across different levels of genetic variation?

**Figure 2.**
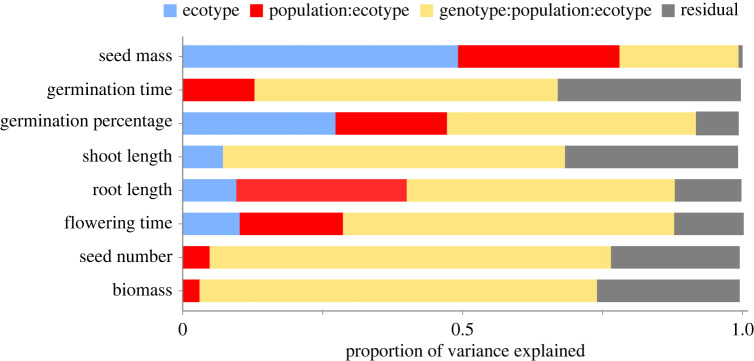
Proportion of variance in seed, seedling, and reproductive traits explained by ecotype, population group, and genotype. Values were calculated from a random effects model parameterized with a nested effect of genotype within population group within ecotype.

We explored the biological level that explained the most phenotypic variation in life-history traits (question 1). Across all traits measured in the greenhouse study, residual variance contributed relatively little (0.7 to 32.7% of variance explained), which indicates that these traits have a strong genetic component. To that end, genotype was the major source of variation in most traits (21.3 to 71.7% of variance explained): genotype explained the most variation in seed number (71.7%) and the least in seed mass (21.3%). Population group within ecotypes was never the largest source of variation in any trait, but still explained appreciable variation in some traits (0 to 30.4% of variance explained). Interestingly, ecotype was only the largest source of variation in one trait, seed mass (49.2%, figure 2). Together with LDA, this suggests that variation in seed mass is strongly associated with ecotypic divergence in *P. hallii*.

### (c) Is climate of origin a good predictor of seed size?

**Figure 3.**
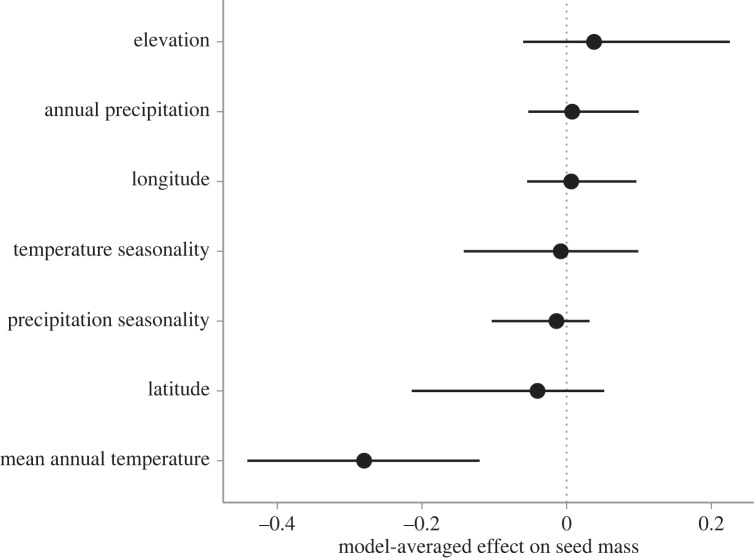
Effect of climate-of-origin variables on seed mass in *P. hallii*. Points are the model-averaged mean effects and error bars represent 95% confidence intervals.

We identified the aspects of climate-of-origin that best explain variation in seed mass in *P. hallii* using model averaging (question 2). Our global model, which included all predictors, explained variation in seed mass well $\left(R_{M}^{2}=0.46\right)$, indicating adequate goodness of fit. Among the best-performing models (ΔAICc < 7), model averaging indicated that only mean annual temperature was a significant predictor of seed mass (*p* < 0.001; electronic supplementary material, table S2, figure 3). Specifically, our model predicted that seed mass would decrease by 0.28 mg for every 1 standard deviation (i.e. 2.34°C) increase in mean annual temperature. No other predictor was significantly associated with seed mass (*p* > 0.25).

### (d) Are there ecotype-specific patterns of covariation between seed size and other life-history traits?

**Figure 4.**
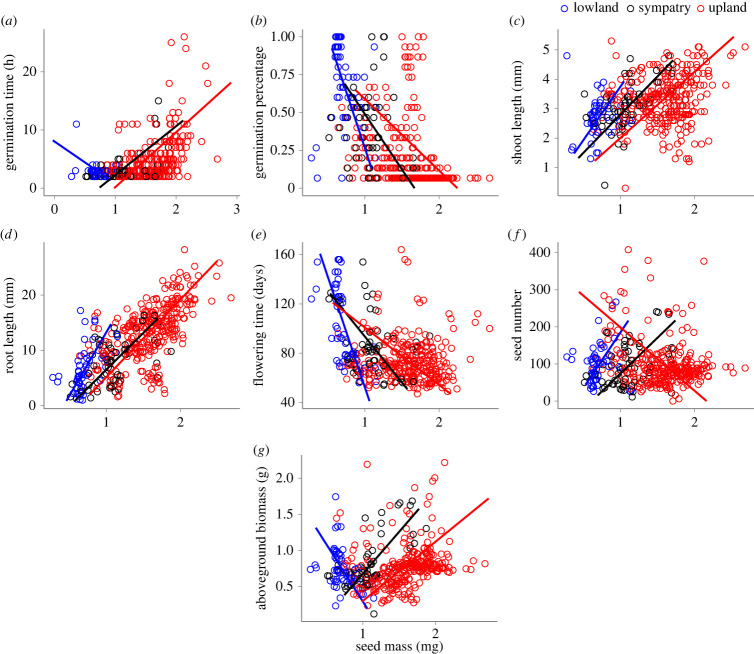
Relationship between seed mass (mg) and (*a*) germination time (hours), (*b*) percentage of seeds germinating, (*c*) shoot length at 18 days after germinating (mm), (*d*) root length at 18 days after germinating (mm), (*e*) flowering time (days), (*f*) the number of seeds produced by each plant, (*g*) aboveground biomass at harvest (mg). All models were performed using standardized major axis regression, including an interaction between traits and ecotypes. Here, points represent genotype-level means.

We determined whether there are ecotype-specific patterns of covariation between seed mass and other life-history traits using standardized major axis (SMA) regression (question 3). Across all comparisons, the relationship between seed mass and other traits differed significantly among ecotypes (*p* < 0.05; electronic supplementary material, table S3). Interestingly, some ecotype-specific patterns existed. The relationship between seed mass and germination time differed among ecotypes: germination time increased significantly with seed mass in the sympatric and upland groups (*p* < 0.001 for each) but decreased non-significantly with increasing seed mass in lowlands (*p* = 0.261; figure 4*a*). Germination percentage decreased with increasing seed mass in the upland (*p* < 0.001) and sympatric (*p* = 0.043) groups (electronic supplementary material, table S4; figure 4*b*).

In the upland and sympatric groups, shoot length (upland, *p* = 0.003; sympatric, *p* < 0.001; figure 4*c*) and root length (*p* < 0.001 for each; figure 4*d*) both increased with increasing seed mass. The lowland group did not show a significant relationship between seed mass and shoot length (*p* = 0.389). But, as with the other groups, root length increased significantly with increasing seed mass (*p* < 0.001; electronic supplementary material, table S4; figure 4*d*). Additionally, a mixed pattern of ecotypic response was observed for the adult and reproductive traits. Seed mass was negatively related to adult biomass in the lowland group (*p* = 0.012), but positively related in the sympatric (*p* < 0.001) and upland (*p* < 0.001) groups (figure 4*g*). Similarly, flowering time decreased significantly with increasing seed mass in both the lowland (*p* < 0.001) and sympatric (*p* = 0.042) groups, but these traits were not significantly related in uplands (*p* = 0.59; figure 4*e*). Finally, seed mass and seed number were negatively associated in uplands and positively associated in the sympatric group (*p* < 0.001 and *p* = 0.005, respectively; electronic supplementary material, table S4; figure 4*f*). The contrasting relationships between seed mass and other life-history traits across ecotypes suggest that selection may favour different trait combinations in different habitats.

### (e) Are ecotypes locally adapted to sites that represent their xeric and mesic origins?

**Figure 5.**
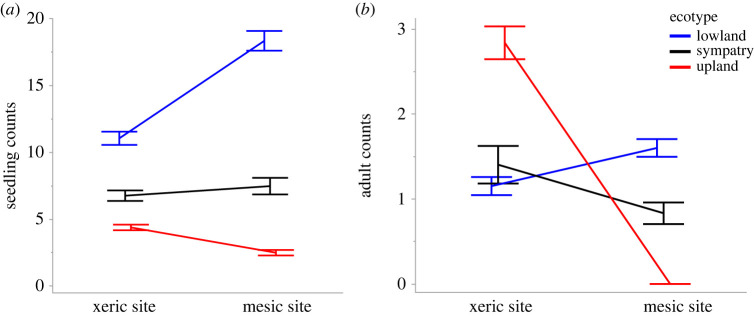
(*a*) Seedling and (*b*) adult recruitment at two sites that represent typical mesic and xeric *P. hallii* habitats. For each panel, lowland is coloured blue, sympatric group is coloured black, and upland is coloured red. Means and confidence intervals were calculated from a Poisson GLM. Error bars represent 1 standard error.

We added seeds of a subset of genotypes at two contrasting field sites to test whether ecotypes are locally adapted to sites that represent their xeric and mesic origins (question 4). There was a significant ecotype-by-site interaction for both seedling and adult recruitment (*p* < 0.0001 for both; table 1). Consistent with local adaptation, lowlands and uplands each had greater seedling recruitment in their own habitat: the lowland group had 38% higher seedling recruitment in the mesic site compared to the xeric site, while the upland ecotype had 37% higher seedling recruitment at the xeric site than at the mesic site (*p* < 0.001). Recruitment for the sympatric group did not differ between habitats (*p* = 0.2207; electronic supplementary material, table S5; figure 5*a*).

**Table 1.** Results of generalized linear models (GLM) on seedling and adult recruitment in different habitats. In the factorial model, ecotype, site and their interaction have been tested and described in the table.

recruitment stage	source of variation	d.f.	*χ^2^*	*p*
seedling	ecotype	2	1162.58	<0.0001
	site	1	63.56	<0.0001
	site × ecotype	2	180.19	<0.0001
adult	ecotype	2	106.76	<0.0001
	site	1	170.03	<0.0001
	site × ecotype	2	345.10	<0.0001

As with seedling recruitment, lowlands and uplands had higher adult recruitment in their respective habitats. Lowland plants recruited 28% more adults at the mesic site than at the xeric site (*p* = 0.007). Uplands showed even stronger evidence of local adaptation: at the xeric site, three adults per plot recruited, while there was no recruitment at the mesic site (*p* < 0.001; electronic supplementary material, table S5; figure 5*b*). The sympatric group had 40% higher adult recruitment at the xeric site than at the mesic site (*p* = 0.013). Overall, this indicates that early seedling recruitment favoured lowlands over uplands at both locations. Later, adult recruitment favoured uplands in xeric habitat and lowlands in mesic habitat.

### (f) How strong is the selection on seed size in xeric and mesic habitats?

**Figure 6.**
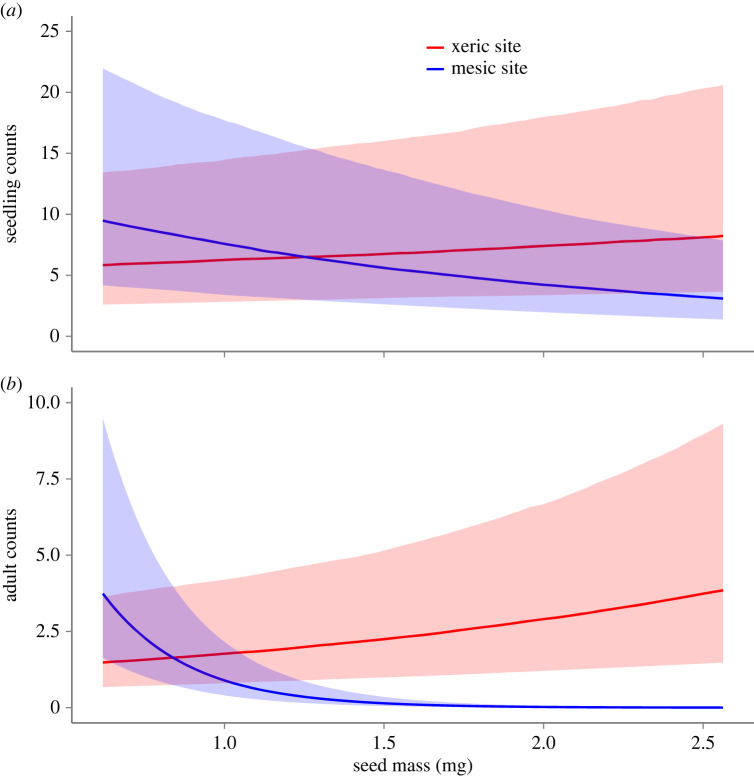
The influence of seed mass on (*a*) seedling and (*b*) adult recruitment at two sites that represent typical mesic and xeric *P. hallii* habitat. For each panel, xeric and mesic sites are coloured red and blue, respectively. Shaded area corresponds to the 80% credible intervals.

We further investigated the strength of selection on seed mass in different field environments (question 5). Consistent with local adaptation, seedling recruitment declined with increasing seed mass at the mesic site (*β* = −0.56, 95% CI = −0.91 to −0.21); at the xeric site, the relationship between seedling recruitment and seed mass did not differ from zero (*β* = 0.20, 95% CI = −0.17 to 0.53). Moreover, the relationship between seed mass and seedling recruitment was significantly more negative at the mesic site than at the xeric site (*β*_lowland–upland_ = −0.75, 95% CI = −0.95 to −0.56, figure 6*a*). Seed mass had a qualitatively similar effect on adult recruitment. At the mesic site, after accounting for seedling number, adult recruitment declined precipitously with increasing seed mass (*β* = −6.62; 95% CI = −8.12 to −5.1). Conversely, at the xeric site, there was a 94% probability that seed mass was negatively related to adult recruitment (*β* = −0.49, 95% credible interval = −1.04 to 0.02). Again, this relationship was substantially more negative at the mesic site than at the xeric site (*β*_lowland–upland_ = −6.13; 95% CI = −7.61 to −4.66, figure 6*b*). Overall, these results indicate that variation in seed mass is an important determinant of establishment in different habitats and could explain the ecological characteristics of *P. hallii* across its geographical range.

## 4. Discussion

Local adaptation is an important process by which natural populations optimize their growth, survival and reproductive strategies to local conditions [22,50]. Our study suggests that local adaptation could explain the divergent patterns of covariation between seed size and other life-history traits across *P. hallii* ecotypes. This pattern may occur when different environments favour opposing trait combinations. In the field, selection on seed size differed dramatically between sites, which is also consistent with strong ecotypic divergence in *P. hallii*. Overall, our study provides the basis for understanding seed-based life-history traits in *P. hallii* and the effect of different seed size strategies for adaptation in xeric and mesic habitats. A brief overview of the research questions and major findings from this study has been shown in table 2.

**Table 2.** A brief overview of research questions, data and main findings.

research conducted	data collected	questions asked	observation
greenhouse experiment	seed mass, seedling, and reproductive traits	how is phenotypic variation in life-history traits arrayed across different levels of genetic variation?	genetic variation significantly explained most of the phenotypic variation across life-history traits
		is climate of origin a good predictor of seed size?	seed mass decreased significantly with increasing temperature of genotype origin
		are there ecotype-specific patterns of covariation between seed size and other life-history traits?	ecotype-specific covariation existed between seed mass and some life-history traits; for example, germination percentage decreased significantly with increasing seed mass for the upland ecotype
reciprocal field experiment	seedling and adult recruitment	are ecotypes locally adapted to sites that represent their xeric and mesic origins?	the establishment of lowland and upland genotypes was favoured in their respective home sites
		how strong is the selection on seed size in xeric and mesic habitats?	variation in seed mass was an important determinant of establishment in different habitats, suggesting that this trait is under strong selection pressure

### (a) Phenotypic variation in multivariate space suggests greater differentiation between ecotypes

The lowland and upland ecotypes in *P. hallii* vary significantly in morphology, physiology and life-history traits [34,35,37,51]. Studies that have investigated this ecotypic divergence were mostly performed with one representative upland genotype (HAL2/ var. *hallii*), one representative lowland genotype (FIL2/ var. *filipes*) and a recombinant population generated from their cross [34–36,51]. A few studies have gained additional insight into *P. hallii* divergence by using a broader range of natural diversity [37,38]. Our study builds upon this work to examine a larger collection of genotypes. Our results demonstrate clear phenotypic distinctions between *P. hallii* populations derived from xeric and mesic habitats, often related to variation in seed mass. This is consistent with evidence from other species [52–54].

### (b) Variation caused by the genetic components and climate of origin on life-history traits

Previous studies with natural populations of *P. hallii* detected genetic differentiation between ecotypes as well as strong population structure within ecotypes. The genetic divergence within ecotypes was significantly correlated with ecoregion, suggesting that local conditions played a significant role in shaping the genetic diversity in *P. hallii* [37,38]. However, the amount of phenotypic variance in life-history traits explained by genetic variation was previously unknown. Our data showed that ecotype explained the most variance in seed mass, which is consistent with some other species [55]. Because *P. hallii* ecotypes co-occur in only a small portion of their geographical range [37,38], seed size and dormancy may be important drivers of establishment in habitats that differ in soil water availability. In a recent study in *P. hallii*, these traits were negatively genetically correlated, which suggests that a trade-off between the two may be important for local adaptation to xeric and mesic habitats [36]. Despite the importance of seed mass for ecotypic divergence and its strong relationship with many other life-history traits, genotype identity, which was nested within ecotype, explained the most variation in most other life-history traits, including seed number and aboveground biomass. This suggests that not all life-history traits contribute in the same manner to local adaptation.

Climate of origin was a major predictor of seed size in *P. hallii*. In particular, seed mass declined with increasing mean annual temperature. Contrary to this result, in *Pinus contorta* and 34 perennial *Glycine* spp., seed size increased with increasing temperature [56,57]. The positive relationship between temperature and seed size seen in these studies is hypothesized to result from basic metabolic requirements—species adapted to higher temperatures would require larger seed mass to convert seed reserves into seedling growth [58,59]. However, seed size decreased with increasing temperature in the Australian perennial grass *Austrodanthonia caespitosa* [60]. This suggests that temperature may be one of several factors that can shape variation in seed size. In *P. hallii*, different seed size strategies might evolve due to tolerance–fecundity trade-offs, where species with larger seeds evolve to tolerate stressful (e.g. dry) conditions and species with smaller seeds evolve to establish in a competitive habitat due to their greater fecundity [61]. The ecological foundation of a tolerance–fecundity trade-off indicates that the observed pattern of seed mass in *P. hallii* may be driven less by temperature *per se* than by some combination of climate and habitat characteristics [62,63].

### (c) Ecotypes had opposing patterns of covariation between seed size and other life-history traits

Environmental factors often select for different patterns of resource allocation to reproductive and vegetative functions to optimize life-history strategies [64,65]. Thus, ecotypes may differ in the pattern of covariation between seed size and other life-history traits. In this study, we often observed significantly different relationships between seed mass and other traits for the lowland and upland groups. But the opposing relationships were rarely statistically significant within both ecotypes. For example, the relationship between seed mass and aboveground biomass was significantly different in the upland and lowland groups (i.e. their slopes differed). The upland ecotype showed a significant negative relationship between seed mass and seed number, but the lowland ecotype showed a non-significant positive relationship. One limitation to interpreting these results is that we had very different statistical power to detect effects in different ecotypes. Thus, we were much more likely to detect a relationship in the upland group, which comprised many genotypes, than in the lowland group. As such, a non-significant effect in the lowland group was less likely to be a true negative than in the upland group. Another limitation of this greenhouse study was that every plant was given the same resources (e.g. water, light, soil). Additionally, because we only examined one bout of reproductive traits in a perennial grass, differences in allocation to current and future reproduction could alter our conclusions over the full lifespan of these plants. Hence, it is difficult to predict whether these relationships reflect those found in the natural conditions under which selection would act. Despite these limitations, this result supports other empirical studies and meta-analyses showing that plant size can covary with seed mass [66,67].

### (d) Ecotype establishment at different sites is constrained by variation in seed size

Two processes are often proposed to explain why particular ecotypes or species only occur in certain habitats: seed limitation and site limitation. Seed limitation occurs when plants are unable to disperse to a site, while site limitation occurs when local conditions—either abiotic or biotic—are unsuitable for the plants [68,69]. Our field study showed an interesting pattern of recruitment limitation for lowland and upland genotypes. Lowland genotypes were able to recruit at both sites, while upland genotypes failed to establish at the mesic site. These results suggest that the establishment of the upland ecotype at the mesic site was constrained by the interaction of seed and site limitations, while the establishment of the lowland ecotype at the xeric site was purely due to seed limitation. These conclusions would not be obvious from focusing on seedling establishment alone. Rather, they can only be drawn by examining the full life-history of *P. hallii*.

Although local adaptation is often invoked as a response to abiotic conditions, the biotic environment may also play an important role [70,71]. For instance, because upland *P. hallii* takes longer to germinate, their seeds may be easier targets for seed predators [72,73]. In these open environments, where large seeds are exposed to predation for long periods, the benefits of producing large seeds must balance or outweigh the costs [74]. On the other hand, the mesic habitat in our field experiment had denser vegetation and more consistent precipitation. In this environment, producing many small seeds that germinate rapidly is often advantageous. This strategy increases the probability that at least some seeds will find microsites suitable for establishment [74,75]. In another study in *P. hallii*, local biotic conditions also played a significant role in establishment (S.R. & T.E.J. 2021, unpublished data). Specifically, upland establishment in a mesic site was only possible when all aboveground vegetation was removed, highlighting the differences in life-history strategies between ecotypes.

## 5. Conclusion

By combining laboratory- and field-based approaches, we show the importance of seed-based life-history traits for *P. hallii*. Briefly, our study showed how genetic divergence influenced phenotypic divergence of life-history traits*.* First, ecotypic divergence was the major source of variation in seed size (question 1). We also found seed mass decreased significantly with increasing temperature of genotype origin (question 2). Additionally, we discovered some significant ecotype-specific relationships between seed mass and other life-history traits, suggesting that *P. hallii* ecotypic divergence is related to seed mass (question 3). Together, these life-history trait relationships can affect seedling establishment and may also affect population dynamics. We found that the establishment of lowland and upland genotypes was favoured in their respective home sites (question 4). This effect may have been driven by differences in selection on seed size (question 5). This provides evidence for the importance of seed traits in local adaptation to habitats that differ greatly in abiotic stress. This also suggests an important link between early life-history strategies and broader patterns of ecotypic divergence in widespread plant species.

## Data Availability

All data collected for this study have been provided in the electronic supplementary material [76].
